# Optimizing cerebral perfusion and hemodynamics during cardiopulmonary bypass through cannula design combining in silico, in vitro and in vivo input

**DOI:** 10.1038/s41598-021-96397-2

**Published:** 2021-08-18

**Authors:** Kristin Hugenroth, Ralf Borchardt, Philine Ritter, Sascha Groß-Hardt, Bart Meyns, Tom Verbelen, Ulrich Steinseifer, Tim A. S. Kaufmann, Ulrich M. Engelmann

**Affiliations:** 1 Department of Cardiovascular Engineering, Institute of Applied Medical Engineering, Faculty of Medicine, RWTH Aachen University, Aachen, Germany; 2 Enmodes GmbH, Aachen, Germany; 3 Department of Cardiac Surgery, University Hospitals Leuven, Leuven, Belgium; 4 Department of Medical Engineering and Applied Mathematics, FH Aachen University of Applied Sciences, Jülich, Germany

**Keywords:** Preclinical research, Computational models, Cardiovascular biology, Biomedical engineering

## Abstract

Cardiopulmonary bypass (CPB) is a standard technique for cardiac surgery, but comes with the risk of severe neurological complications (e.g. stroke) caused by embolisms and/or reduced cerebral perfusion. We report on an aortic cannula prototype design (optiCAN) with helical outflow and jet-splitting dispersion tip that could reduce the risk of embolic events and restores cerebral perfusion to 97.5% of physiological flow during CPB in vivo, whereas a commercial curved-tip cannula yields 74.6%. In further in vitro comparison, pressure loss and hemolysis parameters of optiCAN remain unaffected. Results are reproducibly confirmed in silico for an exemplary human aortic anatomy via computational fluid dynamics (CFD) simulations. Based on CFD simulations, we firstly show that optiCAN design improves aortic root washout, which reduces the risk of thromboembolism. Secondly, we identify regions of the aortic intima with increased risk of plaque release by correlating areas of enhanced plaque growth and high wall shear stresses (WSS). From this we propose another easy-to-manufacture cannula design (opti^2^CAN) that decreases areas burdened by high WSS, while preserving physiological cerebral flow and favorable hemodynamics. With this novel cannula design, we propose a cannulation option to reduce neurological complications and the prevalence of stroke in high-risk patients after CPB.

## Introduction

Cardiopulmonary bypass (CPB) is a standard procedure in cardiac surgery^1,2^. The distal ascending aorta is primarily chosen for cannulation, positioning the cannula close to the origin of the brachiocephalic trunk^2,3^. However, CPB is accompanied by a high incidence of neurological complications^4^, among which stroke is a major factor^5,6^: Generally, a 6% prevalence of stroke after cardiac surgery is reported^7^, of which 2–3% are associated specifically with CPB^8,9^. This number increases remarkably towards 13% in high-risk patients^10^.

Among the main reasons for stroke are diminished brain perfusion, caused by impaired cerebral autoregulation (CA)^11^, and embolisms of released atherosclerotic plaques. CA describes the ability of the brain to stabilize the cerebral perfusion on healthy levels (80–150 mmHg)^12^ under different blood pressures and cardiac stress situations. An intact CA regulates cerebral perfusion via vasodilation and vasoconstriction depending on the cerebral metabolic demand and thereby prevents ischemia as well as hyperemia^13^. However, the CA mechanism is impaired in up to one out of five patients during CPB^14,15^. Reasons for this are assumed to be a decreased cerebral perfusion^16^ in combination with hemodilution and hypercapnia^17^ as well as the rewarming phase during CPB^18^. Patients suffering from impaired CA are generally more vulnerable to perioperative stroke^16,19^, therefore further increasing the number of high-risk patients. Further risks arise from embolism, either caused by thrombosis or the detachment of atherosclerotic plaque due to jet flow from the cannula on the aortic arch intima, which can dislodge atherosclerotic debris^20^.

Impaired CA and release of atherosclerotic plaque during CPB are both related to altered flow conditions in the aortic arch caused by the CPB outflow cannula. To avoid these two major complications, previous studies aimed to optimize the flow-pattern induced by the aortic outflow cannula. Two main approaches were taken: restoration of the naturally helical flow pattern in the aortic arch by inducing a helical outflow^21–23^ and deceleration of the outflow by dispersing the outflow jet^20,22,23^.

Here, we present an outflow-optimized cannula design (optiCAN), which was improved using computational fluid dynamics (CFD) simulations, prototyped and tested in vitro as well as in vivo. Our approach merges the suggestions mentioned above to optimize the aortic arch flow pattern: A helix in the cannula body ensures helical flow in the aortic arch and a flow-optimized cannula tip as well as an additional side hole at the tip decelerates the outflow. Both design features are suggested to increase brain perfusion during CPB. Furthermore, the side hole generates a flow in the ascending aorta, which may prevent possible stagnation regions. Based on in silico predictions, we finally present a further design optimization with manufacturing advantages.

## Results and discussion

**Figure 1:**
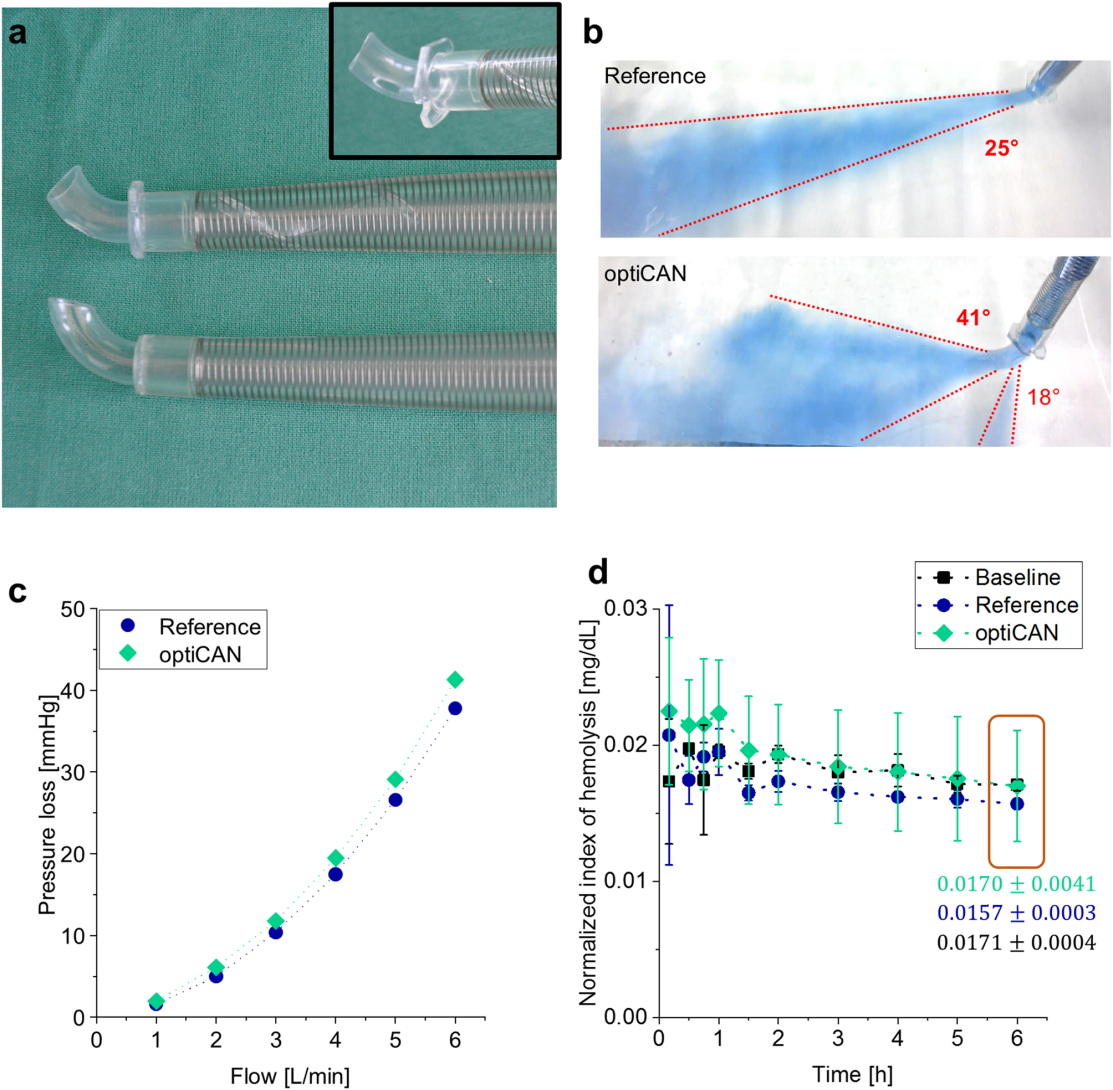
In vitro testing results comparing optiCAN to reference. (**a**) Photography of optiCAN design (upper cannula) in comparison to common reference cannula (lower cannula); both 24 Fr. The inset highlights the optiCAN tip design with outflow side hole. (**b**) Ink-stained outflow-jet visualization at 6 L/min for optiCAN design and reference. The outflow contours are marked with red-dashed lines, which engulf the outflow angle. Note the second outflow jet for the optiCAN design at the side hole with a smaller outflow angle. (**c**) Pressure loss over cannula flow comparing optiCAN design with the reference. (**d**) Normalized index of hemolysis (NIH) measured over six hours comparing a baseline circuit without cannula, to a reference cannula and optiCAN circuits. The NIH values after 6 h are marked (orange box) and explicit values are given for comparison.

With in vitro, in vivo and in silico tests, we compared the optiCAN design, which includes an innovative tip and a helical flow inducing inner wall geometry, with a commonly used curved tip aortic cannula (henceforth called reference). Both cannulas had a 24 Fr diameter and the same shaft length. A photography of both cannula designs is shown in Fig. 1a.

The following sections are divided in three fields of analysis: First, we discuss the results from in vitro testing, followed by those from in vivo testing and then present in silico results from numerical simulations based on the former two analyses. From the synopsis of all three analyses, we discuss limitations and future perspectives in the last section.

### In vitro testing

In vitro testing comprised qualitative outflow jet analysis, pressure loss analysis and hemolysis testing following ASTM and ISO standards with respect to sampling frequency and hematocrit values^24,25^. Details on each procedure are listed in the “Methods” section. The in vitro testing results are summarized in Fig. 1.

The results of qualitative cannula flow are depicted in Fig. 1b and demonstrate a widening of the outflow jet at the cannula tip: the dispersion angle increases by 64% from 25° for reference cannula to 41° for optiCAN design. Additionally, the outflow jet at the hole, intended for additional flow deceleration and aortic root washout (cf. Fig. 3a), yields a dispersion angle of an additional 18°. Under the assumption that these angles serve as indicators for the dispersion capability of both cannulas, the optiCAN design generates a twice as high dispersion compared to the reference cannula. Such increased diffusion lowers the outflow velocity as confirmed in silico by CFD simulations for a cannula design with a similar inner wall geometry^21^ as well as in vitro by particle imaging velocity (PIV) measurements on dispersion-tip cannulas^26^. Furthermore, such increased dispersion will make optiCAN less susceptible to positioning effects and misplacement during CPB in comparison to other curved tip cannulas, which could simplify cannulation handling during surgery (s. in vivo testing, below).

The pressure loss across the cannula rises similarly with increasing flow rates for both the optiCAN design and the reference cannula, as depicted in Fig. 1c. However, the optiCAN design shows a slightly higher pressure loss at all flow rates, reaching just over 40 mmHg at 6 L/min (reference: 38 mmHg). From the two design modifications, helix and tip design, it can be assumed that the former increases, while the latter decreases pressure loss compared to the reference cannula. Thus, both effects presumably counterbalance each other in the optiCAN and lead to similar pressure loss values for both cannulas. Furthermore, with a maximum pressure loss of about 40 mmHg at 6 L/min, optiCAN can compete with other angled dispersion-tip cannulas: its pressure loss is comparable to that of Optiflow™ (LivaNova®; approx. 40 mmHg^27^) and well below those of EZ Glide® (Edwards Lifesciences®; approx. 60 mmHg^28^) and Soft-Flow™ (Medtronic®; 62 mmHg^29^).

Normalized index of hemolysis (NIH) results from hemolysis testing are averaged over 2 independent examination days (s. Supplementary Information S1). After initial fluctuations in the first 1.5 h of testing, the NIH values stabilizes between 0.015 mg/dL and 0.02 mg/dL for all three setups, as seen from Fig. 1d. Generally, the NIH values of all circuits are comparable with each other within their standard errors: The high variances for optiCAN reflect higher uncertainty in NIH measurements, presumably caused primarily by the blood pump and components like connectors and clamps, as seen from the NIH value for baseline measurements. This is in line with previous studies, which report an NIH value of 0.03 mg/mL for the pump used in the present study^30^. Therefore, the hemolytic potential for both cannulas can be considered equivalent. For a quantitative comparison, we use the stabilized NIH values at $t=6$ h, confirming equivalent NIH values for reference and optiCAN design (s. marked values in Fig. 1d). From comparing these values, we derive that optiCAN does not induce additional hemolytic risks compared to the reference cannula within our experimental setup.

In summary, the in vitro testing shows that the optiCAN design reproduce the performance and hemocompatibility of a common reference cannula (cf. Fig. 1c,d), i.e. no alteration of hemolysis for optiCAN compared to the reference. In addition, the optiCAN tip design and side hole increase the dispersion of the cannula outflow compared to the reference cannula (cf. Fig. 1b).

In vitro experiments were furthermore complemented by in vivo tests to incorporate the biological mechanism of a living system.

### In vivo animal trial

**Figure 2:**
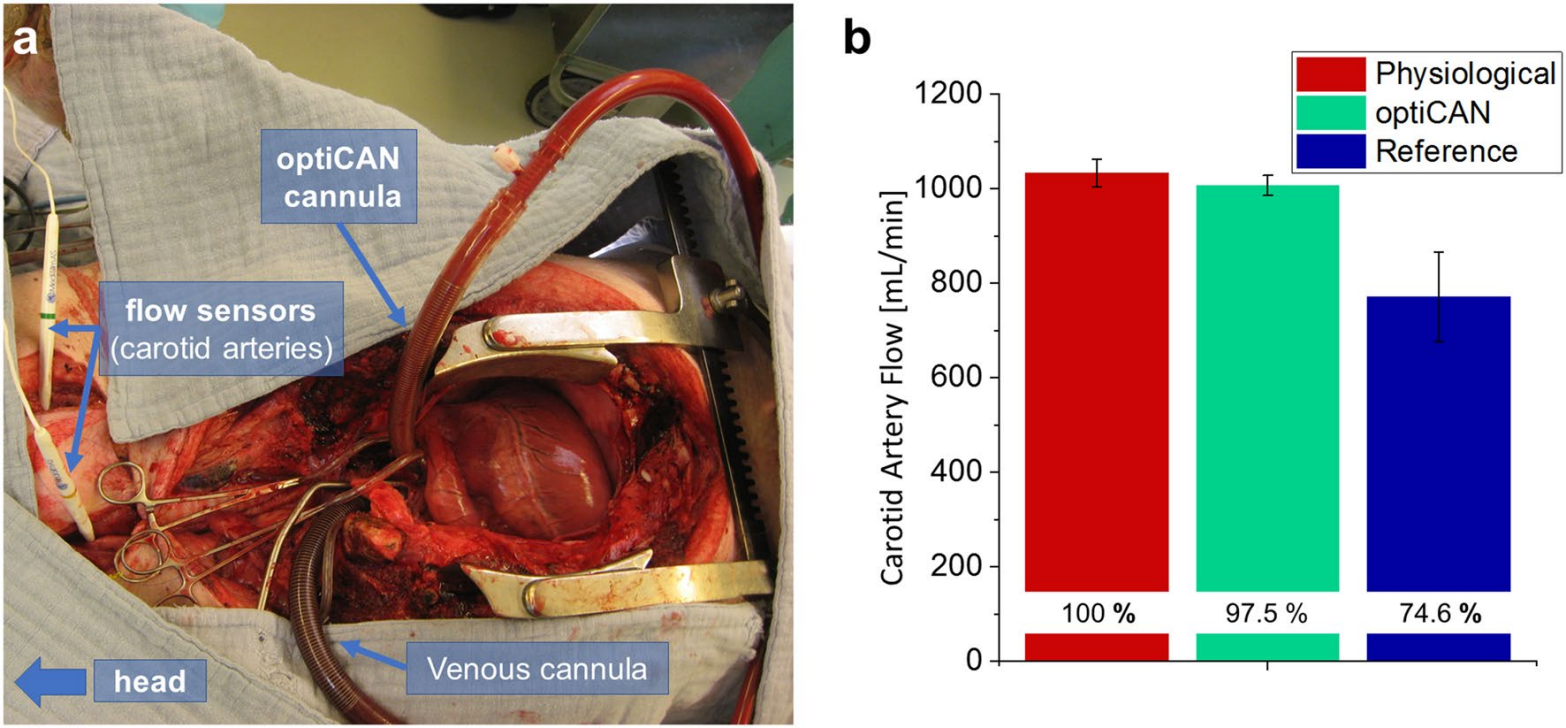
In vivo animal trial in swine during CPB. (**a**) The cerebral flow through the carotid arteries was measured using two flow sensors. (**b**) Comparison of the absolute carotid flow provided by reference and optiCAN cannula and the physiological flow without CPB.

In vivo tests were carried out in swine to measure the carotid artery flow provided by reference and optiCAN cannula during CPB (s. Fig. 2a). Carotid flow serves as a proxy for the cerebral perfusion as it accounts for the majority of the cerebral flow and effects, which increase carotid flow, generally also increase vertebral artery flow^31^. The physiological carotid flow under normal heart activity was measured as ${Q}_{0}=(1033\pm 30)$ mL/min. Using the reference cannula under CPB, the perfusion dropped to ${Q}_{R}=(771\pm 94)$ mL/min (74.6% of physiological value ${Q}_{0}$), as shown in Fig. 2b. In contrast, when using the optiCAN cannula, the carotid flow was almost unchanged under CPB measuring ${Q}_{\mathrm{opti}}=(1007\pm 21)$ mL/min (97.5% of ${Q}_{0}$). In both cases, the cannula flow was equal to the previous cardiac output of 5.16 L/min. These results confirm our assumption that the restoration of the helical aortic arch flow pattern and the deceleration and increased dispersion of the outflow preserve physiological flow in the carotid arteries. The findings from the animal trial are consistent with previous results from in silico^22^ and in vitro studies^23^.

It is well known that a decreased cerebral blood flow during CPB increases the risk of postoperative neurological complications (PNC) in general and of ischemia in particular^13,32^. Assuming a linear relationship between cerebral blood flow and brain oxygenation (in first approximation)^33^, this would correspond to a cerebral oxygen desaturation of 25% during CPB from the drop in CBF for the reference cannula, which in turn is well known to again increase the risk of PNC caused by hypoperfusion^34^. From clinical experience, we know that the majority of patients treated with CPB are above 60 years, many of which have cardiovascular comorbidities (e.g. variable degree of stenosis of their carotid arteries, ranging from 20 to 80%), making them especially vulnerable to cerebral hypoperfusion during CPB. For these high-risk patients a restoration of cerebral perfusion towards physiological level is especially valuable.

However, as we are aware of the anatomic differences between swine and humans and of the individuality of each anatomy, we discuss the applicability of our findings from in vivo testing for clinical application in humans in the subsequent section.

### Numerical simulations

As an established and versatile proof-of-concept method, CFD simulations are employed here to answer three crucial questions for advancing optiCAN technology towards clinical application with reduced risks during CPB:

1. Within which limits can the results from in vivo tests in swine be applied to humans?
2. What risks of embolism from thrombus formation or plaque detachment during CPB are yet to be expected? In other words: How does optiCAN perform regarding hemodynamics, in particular regarding potential regions of stagnating blood flow or regarding the impact of the outflow jet on the aortic arch intima?
3. How can the design be further improved to overcome possible drawbacks regarding hemodynamics and manufacturability?

Question 1 addresses the need for further evidence on the effectiveness of the optiCAN design, before clinical trials in human patients can be arranged. By answering this question, we probe the “Validity of the animal model” presented in “In vivo animal trial”. Question 2 discusses cannula design criteria for evaluating those hemodynamics parameters, which are neither accessible by in vitro nor in vivo tests. Question 3 aims to optimize the current optiCAN design towards safe clinical applicability and cost-effective manufacturability (as a disposable medical device, optiCAN must be able to compete in manufacturing costs). We answer this question in the last subsection by proposing a “Further design optimization”, called opti^2^CAN.

#### Validity of the animal model

**Figure 3:**
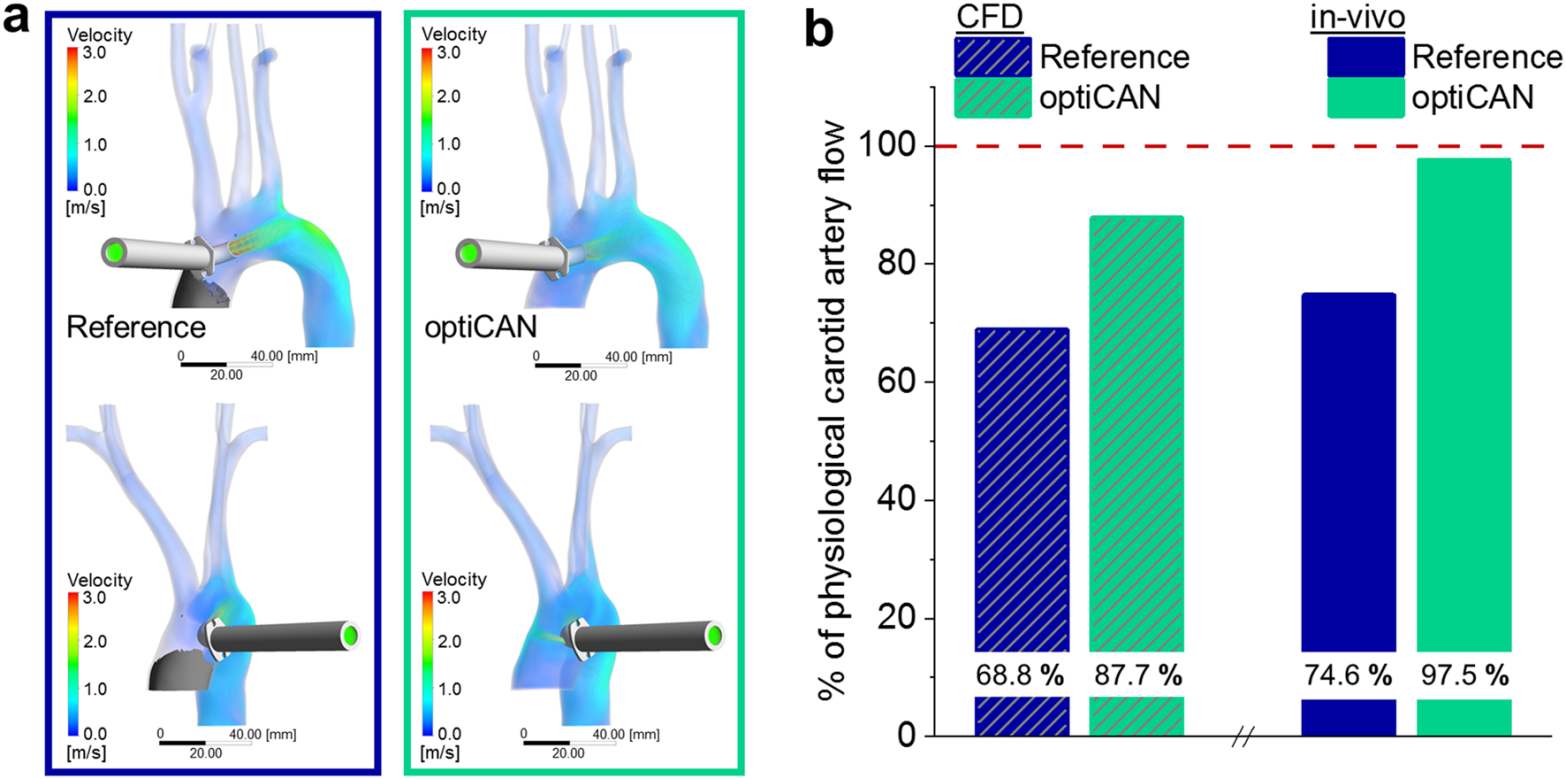
CFD simulation results for a human anatomy. (**a**) Flow field in the aorta and supra-aortic vessels for reference (left) and optiCAN cannula (right) in anterior view (upper) and left lateral view (lower). Gray shades mark the area of low blood flow (velocity below 0.01 m/s). (**b**) Comparison of carotid artery flow (normalized to physiological cerebral flow) comparing reference cannula with optiCAN design from CFD simulation (shaded) and in vivo animal trial. (cf. Fig. 2b). Dashed line marks physiological cerebral flow.

As the aortic arch anatomy of swine differs from human anatomy, we use CFD simulations to investigate the blood flow and distribution in an MRI-derived human aorta arch (s. “Methods” section for details). As shown in Fig. 3a, the streamlines of the cannula outflow in the aortic arch show the helical flow induced by the optiCAN design, as well as the rear flow through the additional hole. Both effects are absent for the reference cannula. While the pattern caused by the optiCAN inflow shows clear helical features from the insertion location onwards, the reference cannula ejects a jet flow, which passes the supra-aortic branches and hits the intima just distal of the left subclavian artery junction. Hence, optiCAN better reproduces the physiological helical flow pattern that was identified as beneficial in previous studies^35^.

When quantitatively comparing the cerebral flow simulated for either cannula with each other, the impression is confirmed: As seen in Fig. 3b, carotid artery flow is 87.7% of the physiological value for optiCAN and 68.8% of the physiological value for the reference. Flow in the vertebral artery is preserved better than in the carotid artery by both cannulas. Therefore, both cannulas show a slightly better performance when considering the overall cerebral flow (carotid plus vertebral artery): optiCAN preserves 89.4% of the overall physiological cerebral flow, while the reference reaches 71.2%. This yields an absolute increase by 18.2% (or 25.6% in relation to the reference) of cerebral perfusion from carotid artery flow using optiCAN. An even higher increase in cerebral perfusion is predicted from including vertebral artery flow (s. Supplementary Information S2 for details). It reproduces the same trend as observed in in vivo testing (even though not as pronounced; in vivo: optiCAN: 97.5%, reference: 74.6%; absolute increase of flow by 22.9% or 30.6% relative to the reference). These quantitative differences between in vivo and in silico results were expected based on two main differences between in-vivo test (with swine) and simulation (human): First and foremost, the anatomy of the aortic arch was different: Swine usually have two supra-aortic branches, where humans have three instead. The simulation was performed with a model of a human aortic arch with three branches. Second, a stiff aortic arch wall was modeled in the simulation, whereas it was flexible in the in vivo testing. However, assuming a stiff aortic wall as used in CFD simulations may even better resemble the situation in high risk patients, as a rather stiff aortic wall is expected for elderly patients and those with cardiovascular disease^36^. Despite potential anatomic differences, the CFD simulated predictions can therefore be considered to approximate the human reality, further substantiating the flow optimization effect of optiCAN.

#### Hemodynamic criteria

Regions of stagnating blood flow with velocities v < 0.01 m/s are prone to thrombus formation^37^. This can even occur for well heparinized patients, if the heparin boluses do not advance into the respective areas due to stagnating blood flow. When occurring in the ascending aorta during CPB, this effect may lead to thromboembolism, once the aortic cross-clamp is removed. In order to estimate the risk for this to happen, we identified regions of stagnating flow (velocity v < 0.01 m/s) as depicted in Fig. 3(a, shaded volume). A low-flow region is only observed in the reference cannula and is situated in the ascending aorta. The positioning of the cross-clamp is highly dependent on patient-specific anatomy and the individual surgical situation, and the aortic geometry used in the simulation moreover simplifies the blockage caused by the clamp. Therefore, the results are merely indicative and cannot be interpreted quantitatively. However, it can be noted that the rear flow from the side hole in optiCAN helps to prevent stagnation, as confirmed by the absence of such regions for optiCAN in Fig. 3a.

**Figure 4:**
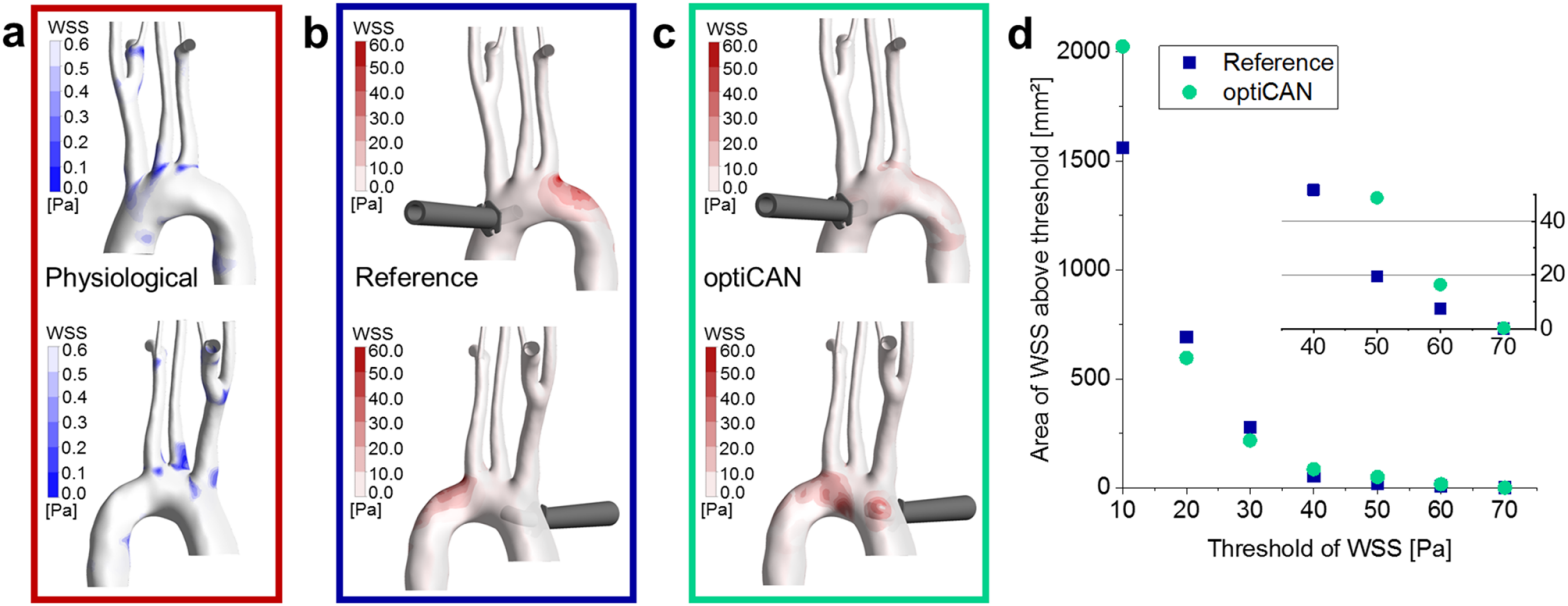
Wall shear stress (WSS) on the intima of the aortic arch. (**a**) WSS during physiological conditions in anterior (upper) and left lateral (lower) view. These can be prone to atherosclerotic plaque formation. (**b**,**c**) WSS during CPB for reference and optiCAN, respectively. High WSS resembles high impact on the intima and can lead to detachment of plaque. (**d**) Accumulated area of WSS above the critical threshold versus the respective critical threshold of WSS for both cannula designs. Inset shows a zoom for high thresholds of WSS.

Wall regions exposed to the high flow velocities of the cannula outflow jet, on the other hand, are prone to the detachment of atherosclerotic plaque during CPB^38^. The impact of the jet on the intima can be quantified by wall shear stress (WSS), with high WSS values occurring in regions of strong impact. The buildup of plaque varies over the aortic arch and is more likely to occur in regions of chronically low WSS^38,39^. Regions of strong impact and those of increased risk for plaque formation under physiological conditions are depicted in Fig. 4. In accordance with Assemat et al., these regions of low WSS in physiological conditions are located at the onset of the supra-aortic branches as well as at the small curvature of the aortic arch^38^ (depicted in Fig. 4a). Under CPB, both cannulas increase the WSS in specific regions depending on their outflow characteristics, which is shown in Fig. 4b,c. Using the reference cannula, the left lateral side of the left subclavian artery junction is affected by high WSS, while using optiCAN it is rather the posterior ascending aorta and posterior distal aortic arch that are affected by high WSS, due to the different tip designs. Thus, the outflow jet of the reference cannula generates high WSS at a location more prone to plaque buildup (left subclavian artery junction; cf. Fig. 4a,b), thereby increasing the risk of plaque detachment. The outflow jets of the optiCAN, on the other side, generate high WSS at locations less prone to plaque formation (posterior aortic arch wall, cf. Fig. 4a,c).

For a quantitative analysis, we calculated the accumulated area of the aortic wall, in which the time-averaged (s. “Methods” section) WSS exceeds a certain WSS threshold. Since it is yet unclear, which WSS values are critical for detachment of atherosclerotic plaque from the aortic intima^40–42^, WSS thresholds were arbitrarily chosen ranging from 10 to 70 Pa, with 10 Pa step size as shown in Fig. 4d. The area of WSS exceeding these threshold value decreases in an exponential fashion with increasing threshold value for both cannulas. For WSS thresholds of 20 and 30 Pa, the reference cannula shows a larger accumulated area of WSS exceeding that threshold. For WSS thresholds of 40 Pa and above on the other hand, optiCAN shows a larger area with WSS above the threshold. The situation is yet different for the smallest threshold value of 10 Pa, where the area for optiCAN surpasses that for the reference, which results from the widened dispersion angle of optiCAN including the side hole outflow (cf. Fig. 1b) that naturally increases low WSS impact on the intima.

Such detailed and quantitative hemodynamic considerations complement results for commercially available perfusion-improved arterial cannulas for CPB: For example, pressure loss data are available for the Optiflow® by LivaNova®^27^, Soft-Flow® by Medtronic®^20^ and EZ Glide® by Edwards Lifesciences®^28^ (on the market) as well as the Stealthflow cannula (published in vivo studies^43,44^). An interesting approach was chosen recently by Gennari et al., who report microembolic signals (MES) during CPB in 19 out of 30 patients^45^. The study compares Optiflow® and EZ Glide® cannulas and doesn’t find a significant difference regarding tendency for MES (Optiflow®: N = 10, EZ Glide®: N = 9). The authors furthermore find that MES increase most during the actual aortic cannulation event and the start of CPB as well as opening of the cross clamp. It would be interesting to complement such in vivo examinations with CFD simulations to examine the potential relationship between WSS and MES with special focus on changes in the surgical procedure (i.e. cannulation, start of CPB, etc.).

#### Further design optimization

**Figure 5:**
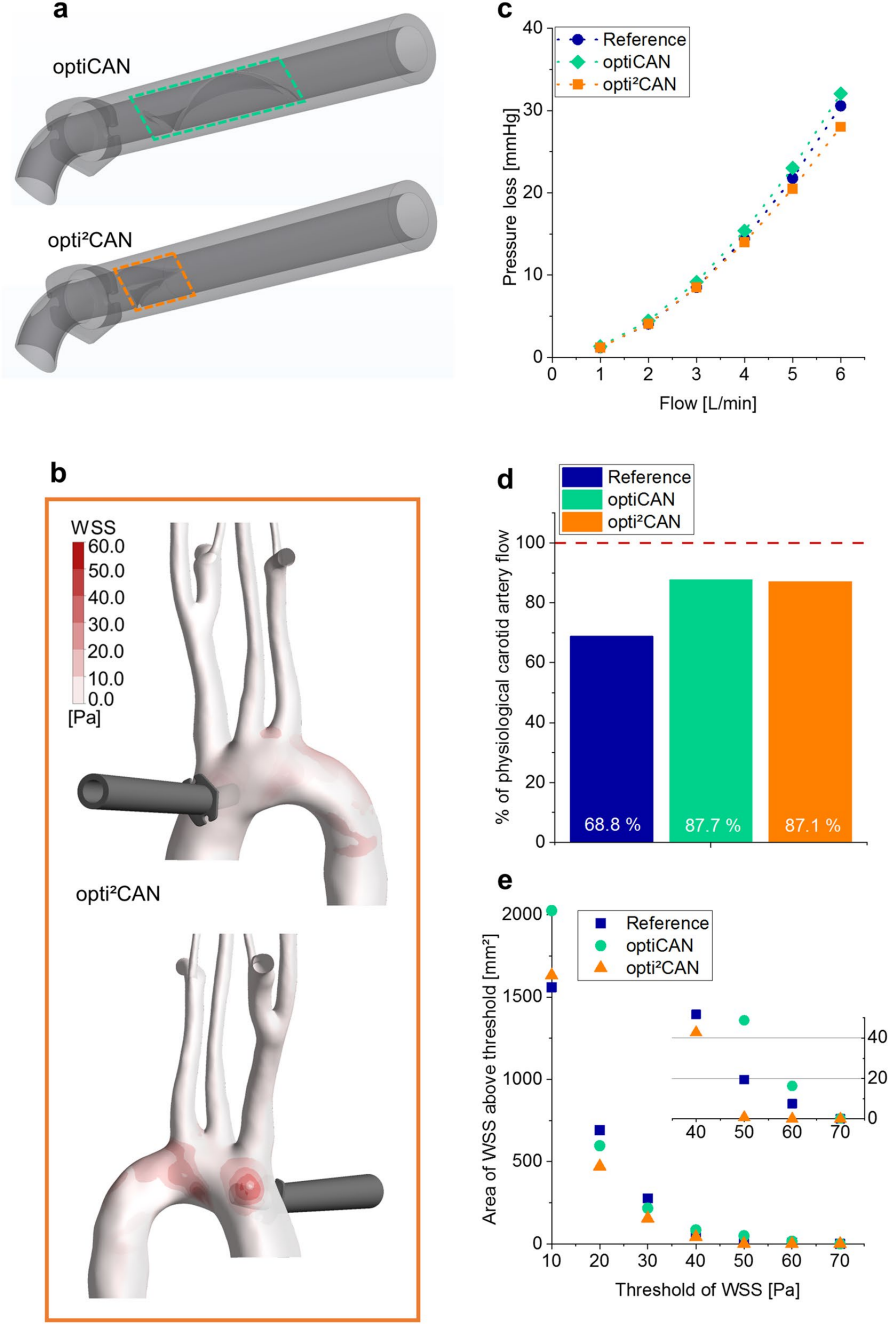
Results for further CFD-optimized design opti^2^CAN. (**a**) 3D models of the optiCAN and opti^2^CAN design. Different inner wall geometry is highlighted with dashed lines. (**b**) WSS on the intima of the aortic arch for opti^2^CAN during CPB. High WSS resembles high impact on the intima and can lead to detachment of plaque. (**c**) Pressure loss over cannula flow predicted from CFD simulations comparing reference, optiCAN and opti^2^CAN design. (**d**) Comparison of relative carotid artery flow for reference, optiCAN and opti^2^CAN design. Dashed line marks physiological level. (**e**) Accumulated area of WSS above the critical threshold versus the respective critical threshold of WSS for all three cannula designs. Inset shows a zoom for high thresholds of WSS.

Based on the results presented in previous sections and prototyping experience, we identified the following design optimizations of optiCAN: (1) lower pressure loss over the cannula (cf. Fig. 1c), (2) lower WSS on the aortic arch intima (cf. Fig. 4d) and (3) competitive (cost-effective) manufacturability of the new cannula design. All of the above under the precondition that the potential optimizations must not compromise on the improved cerebral perfusion. Since the inner wall geometry of the cannula shaft was assumed to cause both an increased pressure loss as well as manufacturing difficulties, the helix was replaced by a shortened double-blade-version (s. Fig. 5a). The flow-splitting and dispersing tip design was retained. CFD simulation results confirmed less pressure loss for opti^2^CAN, (s. Fig. 5c): The pressure loss of opti^2^CAN is constantly below that of optiCAN, presumably due to the shorter helix length. Its pressure loss is furthermore below that of the reference cannula, allowing higher maximum flow rates during CPB. This takes advantage of the decreased pressure loss caused by the dispersion-tip with additional side hole as discussed above (cf. “In vitro testing”). The simulations show the same tendency regarding the difference between pressure loss of optiCAN and reference cannula as the in vitro tests (cf. Fig. 1c). Quantitatively, the discrepancy between in silico and in vitro results regarding the pressure loss difference between the two cannulas is 0.63 mmHg on average. This is probably due to manufacturing inaccuracies, which are not represented in the simulation. Notice furthermore, that the absolute pressure loss values are constantly lower in the simulations, since the shafts of all cannulas were shortened to reduce simulation time.

The cerebral flow provided by opti^2^CAN was predicted in the same way as described in “Validity of the animal model”. As the results in Fig. 5d show, opti^2^CAN performs just as well as optiCAN, restoring 87.1% of the carotid artery flow during CPB. From this, one can assume from CFD analysis that the opti^2^CAN design will preserve physiological flow during CPB equally well as the in vivo tested optiCAN design, thereby restoring physiological cerebral flow and meeting the proposed design precondition.

Concerning the hemodynamic criteria of as low as possible area of high WSS, opti^2^CAN revealed a substantial improvement. The accumulated area of high WSS was constantly below that of both the reference and optiCAN design (Fig. 5b,e). In particular, the area with WSS above 50 Pa effectively decreased to zero (i.e. zero affected area), whereas the same occurs for reference and optiCAN only for WSS > 70 Pa. This shows lower impact on the aortic arch intima and gives reason to assume a decreased risk of plaque detachment during CPB using opti^2^CAN.

Along the way, the manufacturing process was simplified by going from precision gluing the inner helix geometry for optiCAN, to potentially manufacturing opti^2^CAN in one piece by means of injection molding making gluing redundant. This could not only reduce manufacturing time and cost but would also increase reproducibility and simplifies quality control for large scale manufacturing.

#### Limitations and future work

In silico pressure loss results are in good agreement with in vitro pressure loss measurements (cf. Figs. 1c and 5c) and in silico cerebral perfusion results are consistent with those acquired through in vivo testing (cf. Fig. 3b. Overall the cerebral perfusion could be restored to physiological flow with our proposed cannula designs. While acknowledging these findings for advancing safe CBP treatment in general, we are aware of the following limitations of our approach from which we derive future research perspectives as follows:

Albeit good agreement between in vivo results from CPB in swine and in silico results from CFD simulations in a human aorta was achieved, statistical result validation is warranted by systematically repeating our CFD analysis in multiple human anatomies and additional in vitro and in vivo experiments. In this process, prototype testing of the improved opti^2^CAN cannula geometry is also intended. Additionally, porcine aortic arch anatomies could then be extracted from the test animals and subsequently used in CFD simulations to allow for more accurate case-by-case validation of our CFD model.

Measures were taken to ensure identical placement of the cannulas during the animal trial (s. “Methods”). The impact of placement variations (for example angle of the cannula tip relative to the aortic wall and positioning of the cannula relative to the aorta) will be investigated both by additional in vivo as well as in silico investigations^46^. Furthermore, different human aortic anatomies could be analyzed. Both cannula placement and aortic anatomy will most likely influence WSS distribution as well as the perfusion of the supra-aortic vessels, because they have an influence on factors like distance between cannula tip and aortic wall, distance from cannula tip to supra-aortic vessels and angle of the inflow jet relative to the aorta. Furthermore, it is assumed that dispersion-increased designs (like optiCAN and opti^2^CAN) are less susceptible to position effects on cerebral perfusion than regular straight-tip designs—this remains to be validated, however.

For stable patients with negligible pressure changes, constant boundary conditions and constant cannula flow can be considered an adequate approximation for the CPB setting. For a more accurate prediction of physiological flow, transient simulations with pulsatile boundary conditions can be evaluated in future simulations studies.

Finally, optiCAN and opti^2^CAN should be tested against other dispersion-tip cannulas such as Optiflow®, Soft-Flow® and EZ Glide®, in order to assess their suitability for competitive clinical application.

## Conclusion

We analyzed a flow optimizing aortic cannula design (optiCAN) intended for use during CPB. In vivo testing in swine revealed a restoration of cerebral perfusion to 97.5% of the physiological flow during CPB, which is attributed to a helical outflow and dispersion-induced deceleration. The preserved cerebral flow reduces risk of oxygen desaturation during CPB and of PNC after CPB considerably, while pressure loss and hemolysis were not considerably higher compared to reference. Furthermore, the increased dispersion provides easier positioning of the optiCAN during surgery as compared to other curved tip cannulas and decreases the risk of misplacement of the cannula due to patient movement. These results from in vivo can be reproduced in silico for an exemplary human aortic arch employing CFD simulations. Here we showed that the optiCAN jet-splitting and dispersion tip design nullifies blood flow stagnation areas in the ascending aorta, remedying the risk of thrombus formation during CPB. Within the CFD framework we further assessed the risk of embolism during CPB due to plaque detachment by comparing the strength and distribution of WSS on the aortic arch intima: We reveal that areas of high WSS is slightly increased for optiCAN compared to the reference cannula, making both susceptible to plaque detachment potentially causing embolism. We demonstrate via CFD simulations that the area of high WSS as well as pressure loss of the optiCAN design can be reduced by introducing another design optimization (opti^2^CAN), which incorporates a helical-flow-inducing double-blade geometry in the cannula shaft. At the same time, opti^2^CAN preserves physiological flow for cerebral perfusion during CPB in the same way as optiCAN. Thereby, opti^2^CAN design could reduce the prevalence of stroke and embolism in high-risk patients at the same time, while adding improved manufacturability due to simplified assembly. With this we present a novel design option to reduce cannulation side effects during CPB and hope to contribute to improved therapy options for high-risk patients.

## Methods

### Cannulas for in vitro and in vivo experiments

The reference cannula was a 24 Fr curved tip aortic cannula in the style of, for example, PureFlex™, Curved Tip (LivaNova plc, London, Great Britain), DLP™ Curved Tip (Medtronic plc, Dublin, Irland) or Medos angled tip arterial cannula (Fresenius Medical Care Deutschland GmbH, Bad Homburg, Germany). The optiCAN tip and helix were designed based on previous CFD-optimization iterations^22^, but improved by reducing the tip diameter at the insertion site equal to the reference cannula to ensure simple and safe placement. Tip and helix prototypes were manufactured by injection molding and attached to, respectively precision glued into, the wire-enforced silicon cannula body of a commercial standard cannula from which the tip had been removed. This ensured that the cannulas differed only in terms of their tips and the inner wall geometry.

### Ink-stained outflow-jet analysis

For ink-stained jet-flow analysis, the respective cannula was connected via silicon tubing to a pump with adjustable steady flow between 1 and 6 L/min. The outflow cannula was placed in a water reservoir allowing for steady outflow with the tip well under water. At defined time steps, 10 mL of commercially available blue ink (4001 Blue Ink, Pelikan Holding AG, Schindellegi, Switzerland) were added to the inflow to visualize the outflow-jet. The ink-stained outflow-jet was recorded on video (1920 × 1080 pixels at 30 FPS) and analyzed in series of single pictures.

### Pressure loss analysis

For the pressure loss analysis, the cannulas were connected to a centrifugal pump via silicon tubing, while the cannula tip was placed in a water reservoir allowing for steady outflow with the tip well under water. The flow was regulated to steady rates of 1, 2, 3, 4, 5 and 6 L/min, monitored by by SonoTT™ Clamp-On Transducers (em-tec GmbH, Finning, Germany). The pressure was measured at the luer lock connector of the cannula, at approximately 22.2 cm distance to the cannula tip, by Xtrans® pressure sensores (CODAN Medizinische Geräte GmbH & Co KG, Lensahn, Germany).

### Hemolysis analysis

Hemolysis tests were performed with heparinized porcine blood on two independent test days following ASTM and ISO standards: three cannulas (2 × optiCAN, 1 × reference) were tested in parallel, each in an individual blood circuit with approx. 520 mL priming volume. For reference, a fourth circuit without a cannula (baseline) was also examined. Each circuit was operated at (5.12–5.16) L/min by a roller pump (Jostra, formerly Maquet Cardiopulmonary AG, Hirrlingen, Germany), their parts connected by 3/8″ PVC tubing (Raumedic AG, Helmbrechts, Germany) and kept at constant temperature of 37 °C. The hematocrit was determined using a hematology analyzer (Celltac Alpha MEK 6450 K; Nihon Kohden Europe GmbH, Rosbach vor der Hohe, Germany) and the flow rate was measured by a SonoTT ultrasonic flow probe (Em-tec Flow Technology LP, Berkshire, NY, USA) attached to the tubing. Porcine Hematocrit was stabilized at $Ht=\left(12\pm 1\right) \mathrm{g}/\mathrm{mL}$^25^.

4 mL samples were taken following ASTM and ISO standards^24,25^ from the circuits at times $t=(\mathrm{15,30,45,60,90,120,180,240,300,360})$ min, from which the plasma free hemoglobin ($fHb$) was obtained using an Ultrospect 2100pro photometer (GE Healthcare Europe GmbH, Freiburg, Germany). The normalized index of hemolysis (NIH) was determined from the difference in plasma free hemoglobin between two measurements ($\Delta fHb$), the duration between consecutive sampling ($T$) as well as circuit volume ($V$), and flow rate ($Q$):

$$NIH \left(\frac{g}{100 L}\right)= \frac{\Delta fHb}{T}*\frac{V*(100-Ht)}{100*Q}$$

### In vivo animal trial

This study was approved by the KU Leuven animal ethics committee (P157/2011) and was performed in accordance with relevant guidelines and regulations The animal received humane care in compliance with the ‘Principles of Laboratory Animal Care’ formulated by the National Society for Medical Research and the ‘Guide for the Care and Use of Laboratory Animals’ prepared by the Institute of Laboratory Animal Resources (National Institutes of Health). Where applicable, authors complied with the ARRIVE guidelines (only one in-vivo test on one animal was performed).

One swine (66.7 kg, aged 4.2 months) was included in the study and received general anesthesia (intramuscular, tiletamine/zolazepame—8 mg/kg, xylazine hydrochloride—2.5 mg/kg) at the initiation of the procedure. Anesthesia was maintained by continuous administration of propofol (10 mg/kg/h) and fentanyl (0.15 mg/kg/min). After intubation, the animal was mechanically ventilated with the use of a volume-controlled respirator (Cicero, Drägerwerk AG & Co. KGaA, Lübeck, Germany). For rhythm stabilization, animals were primed with 300 mg amiodarone and 0.5 mg/kg/h lidocaine was continuously administered. A protective mechanical ventilation was maintained while on CPB, including pressure-controlled ventilation at an I:E ratio of 2:1, a respiratory rate of minimally 6/min with an FiO_2_ of 0.25, low tidal volume (6 mL/kg body weight) and high PEEP (≥ 10 cmH_2_O). During the entire experiment, phenylephrine was the only vasoactive/inotropic medication allowed to maintain a mean arterial pressure above 50 mmHg.

A median sternotomy was performed and the heart was exposed in a pericardial cradle. After administration of heparin and placement of purse strings on the ascending aorta and right atrium, an arterial cannula was placed in the ascending aorta and a venous cannula in the right atrium and CPB was started. Aortic cannulation was performed first with the reference cannula and then with optiCAN. To ensure that the positioning and tip orientation was the same for both cannulas, they had identical suture flanges and sutures were placed at the same locations. The flow rates at the two carotid arteries were monitored with a 5 mm QuickFit TTFM Flow Probe (Medistim ASA, Oslo, Norway).

### Computational fluid dynamics simulations

Computational fluid dynamics (CFD) simulations were conducted to compare the optiCAN design with a common aortic cannula regarding their influence on the hemodynamics. The hemodynamic parameters analyzed were pressure loss across the cannula, areas of increased wall shear stress, low flow regions and distribution of blood flow to the upper branches of the aortic arch.

Two simulation setups were used: For the pressure loss analysis, the cannulas were placed in a model of a reservoir to mimic the in vitro pressure loss measurements (reservoir setup, (cf. Figs. 1b, 5c),). For the analysis of all other parameters, a model of a human aortic arch with and without cannulas was used (aortic arch setup, cf. Fig. 3). The geometry of the aortic arch was segmented from magnet resonance imaging data of a healthy 28-year-old male volunteer. A clinician confirmed the correct positioning of the cannulas. All geometries were prepared using 3-matic (Materialise NV, Leuven, Belgium).

The simulation meshes were created with Ansys ICEM CFD 2019 R3 (Ansys Inc, Canonsburg, US). Tetrahedral elements plus 14 prism layers with a first layer height of 0.005 mm were used. A mesh sensitivity study was conducted (see Supplementary Information S3).

The CFD simulations were performed using Ansys CFX 2019 R3 (Ansys Inc, Canonsburg, US). The reservoir setup was implemented as a steady state simulation with a constant cannula flow rate (1, 2, 3, 4, 5 and 6 L/min) and ambient pressure boundary condition at the walls of the reservoir. Water with a density of 997.0 kg/m^3^ and dynamic viscosity of 0.8899 mPa*s was used to facilitate the subsequent comparison with in vitro experiments.

For the aortic arch setup, a transient simulation setup with constant boundary conditions was chosen, since the short distance between cannula tip and aortic arch wall led to transient effects in the jet flow. A time step of 0.5 ms and a simulation time of 0.45 s were chosen. Cannula flow was set to 5.16 L/min, which corresponds to the average flow rate during the in vivo experiments. A wall was placed in the ascending aorta to model the aortic cross-clamp and a constant outlet pressure of 90 mmHg and a loss coefficient of 50 were defined for all remaining boundaries. The loss coefficient was deduced from the Darcy-Weisbach equation for friction-related pressure loss in a previous study^22^.

In both reservoir and aortic arch setup, blood was modeled using a shear dependent viscosity model based on Ballyk et al.^47^ (see Supplementary Information S4). A density of 1056.4 kg/m^3^ was chosen, which corresponds to a hematocrit of 44%. In both setups the SST turbulence model was applied, and the convergence criterion was defined by a maximum residuum of ${10}^{-4}$. Additionally, pressure at the cannula inlet (reservoir and aortic arch setup) as well as average WSS and mass flows at the outlets (aortic arch setup) were monitored to ensure stabilized predictions and variations well below 3% over the final 100 time steps.

Post processing was performed using CFD-Post 19.3 (Ansys Inc, Canonsburg, US). The aortic arch setup was analyzed using variables that were averaged over the last 100 time steps.

Pressure loss was defined as the difference between predefined wall pressure of the reservoir and pressure at the cannula inlet, mimicking the experimental setup. WSS values were automatically calculated by the CFD-Post formulation, which is provided in Supplementary Information S5.

Further details of the meshing and simulation setup can be found in the Supplementary Information S3 and S4.

## Supplementary Information

Supplementary Information.

## Data Availability

The data analyzed during the study are available from the corresponding authors upon request.
