# Supplementary material for: Optimizing cerebral perfusion and hemodynamics during cardiopulmonary bypass through cannula design combining in silico, in vitro and in vivo input
Source: Sci Rep. 2021 Aug 18;11:16800. doi: 10.1038/s41598-021-96397-2 (PMC8373878; doi:10.1038/s41598-021-96397-2)
Supplement: Supplementary file 1 — Supplementary Information. [file 41598_2021_96397_MOESM1_ESM.pdf]

Supplementary Information

Optimizing cerebral perfusion and hemodynamics during cardiopulmonary bypass through cannula design using combining in silico, in vitro and in vivo input

Kristin Hugenroth[1,2], Ralf Borchardt[1], Philine Ritter[1], Bart Meyns[3], Tom Verbelen[3], Ulrich Steinseifer[1], Tim A.S. Kaufmann[1,2] and Ulrich M. Engelmann[2,4]

[1] Dept. of Cardiovascular Engineering, Institute of Applied Medical Engineering, Helmholtz-Institute, RWTH Aachen University, Aachen, Germany
[2] enmodes GmbH, Aachen, Germany
[3] Dept. of Cardiac Surgery, University Hospitals Leuven, Leuven, Belgium
[4] Dept. of Medical Engineering and Technomathematics, FH Aachen University of Applied Sciences, Jülich, Germany

S1: Hemolysis datasets

Hemolysis testing was performed on 2 independent measurement days with 4 circuits each: one baseline circuit (with no cannula), one reference cannula, two optiCAN cannulas. We calculated the arithmetic mean according to $\bar{x} = \frac{1}{N}\sum_{i=1}^{N} x_i$ with the total number of input values N and the standard deviation (SD) according to $s = \sqrt{\frac{1}{N-1}\sum_{i=1}^{N}(\bar{x} - x_i)^2}$.

Table S1: Results for hemolysis testing for the first and second measurement day and the mean values.

	Day 1 NIH [mg/mL]				Day 2 NIH [mg/mL]				Mean NIH [mg/mL]					
**Time [h]**	Baseline	Reference	optiCAN 1	optiCAN 2	Baseline	Reference	optiCAN 1	optiCAN 2	Baseline	SD	Reference	SD	optiCAN	SD
**1/3**	0.0141	0.014	0.0225	0.0209	0.0206	0.0275	0.0168	0.0298	0.01735	0.0046	0.02075	0.00955	0.0225	0.00543
**1/2**	0.0197	0.0162	0.0224	0.019	0.0198	0.0187	0.0258	0.0186	0.01975	7.07107E-5	0.01745	0.00177	0.02145	0.00336
**3/4**	0.0146	0.0184	0.0254	0.017	0.0203	0.0199	0.026	0.0178	0.01745	0.00403	0.01915	0.00106	0.02155	0.00481
**1**	0.0192	0.0183	0.0222	0.0245	0.0199	0.0207	0.0258	0.0169	0.01955	4.94975E-4	0.0195	0.0017	0.02235	0.00393
**3/2**	0.0177	0.0161	0.0159	0.0222	0.0185	0.0169	0.0238	0.0166	0.0181	5.65685E-4	0.0165	5.65685E-4	0.01963	0.00396
**2**	0.0189	0.0179	0.022	0.0162	0.0198	0.0168	0.023	0.0161	0.01935	6.36396E-4	0.01735	7.77817E-4	0.01932	0.00369
**3**	0.0171	0.017	0.0194	0.0157	0.0189	0.0161	0.0239	0.0147	0.018	0.00127	0.01655	6.36396E-4	0.01843	0.00417
**4**	0.0173	0.0163	0.0208	0.0144	0.019	0.0161	0.0227	0.0143	0.01815	0.0012	0.0162	1.41421E-4	0.01805	0.00434
**5**	0.0167	0.0165	0.0199	0.0143	0.0176	0.0156	0.0228	0.0132	0.01715	6.36396E-4	0.01605	6.36396E-4	0.01755	0.00457
**6**	0.0168	0.0159	0.0194	0.0145	0.0174	0.0155	0.0214	0.0127	0.0171	4.24264E-4	0.0157	2.82843E-4	0.017	0.00408

S2: In silico datasets

Flow in the vertebral artery is preserved better than in the carotid artery by both cannulas. Therefore, both cannulas show a slightly better performance when considering the overall cerebral flow (carotid plus vertebral artery): optiCAN preserves 89.4% of the overall physiological cerebral flow and the reference preserves 71.2% of the overall physiological cerebral flow. This yields an absolute increase in cerebral perfusion by 18.2% or 25.7% relative to the reference cannula.

Data:

**Location**		**Physiological**	**Reference**	**optiCAN**	**opti²CAN**
**Vertebral artery**	Flow (l/min)	0,149	0,121	0,144	0,146
	% of physiological	100,00%	**81,25%**	**96,69%**	**98,28%**
**Carotid artery**	Flow (l/min)	0,622	0,428	0,546	0,542
	% of physiological	100,00%	**68,75%**	**87,70%**	**87,12%**
**Cerebral flow (vertebral + carotid artery)**	Flow (l/min)	0,771	0,549	0,690	0,688
	% of physiological	100,00%	**71,17%**	**89,44%**	**89,27%**

S3: Meshing

The simulation meshes were created with Ansys ICEM CFD 2019 R3 (Ansys Inc, Canonsburg, US).

The mesh sensitivity study was conducted with the aortic arch setup of the opti²CAN cannula.

		WSS	Mass flow (kg/s)							Pressure (mmHg)			Mesh size	
		**maximum**	**DescAO**	**LCCA**	**LSA**	**LVA**	**RCCA**	**RSA**	**RSA**	**average**	**maximum**	**minimum**	**Elements**	**Nodes**
**absolute value**	**coarse**	39,340	-0,06517	-0,00364	-0,00625	-0,00130	-0,00577	-0,00749	-0,00122	95,766	133,959	66,561	2245848	780473
	**medium**	63,388	-0,06501	-0,00366	-0,00624	-0,00132	-0,00585	-0,00754	-0,00124	95,805	132,579	66,286	11056080	3256452
	**fine**	64,412	-0,06488	-0,00375	-0,00633	-0,00135	-0,00583	-0,00748	-0,00124	95,963	131,978	66,993	13765791	3784569

		WSS maximum	DescAO	LCCA	LSA	LVA	RCCA	RSA	RSA	Pressure average	Pressure maximum	Pressure minimum
**deviation from finest mesh**	**coarse**	38,93%	-0,44%	2,87%	1,20%	3,09%	0,89%	-0,14%	1,84%	0,21%	-1,50%	0,64%
	**medium**	1,59%	-0,20%	2,45%	1,45%	2,13%	-0,35%	-0,86%	0,07%	0,16%	-0,46%	1,05%
	**fine**	*0,00%*	*0,00%*	*0,00%*	*0,00%*	*0,00%*	*0,00%*	*0,00%*	*0,00%*	*0,00%*	*0,00%*	*0,00%*

We selected the settings of the medium mesh for the further simulations. These settings include:

- Maximum element size: Aortic wall: 2 mm; cannula body: 0.5 mm; cannula tip: 0.25 mm
- Curvature refinement: 20x with minimum size limit of 0.0625 mm
- Prism layers: 14 layers on cannula and aortic wall, initial height 0.005 mm, height ratio 1.3
- Meshing method: Fluent Meshing (expansion factor: 1.05) on Octree surface mesh

S4: Blood viscosity model

To account for the non-Newtonian rheology of blood, we used the blood model described by Ballyk et al. (1994), which describes the non-Newtonian viscosity μ_{NN} based on the shear rate γ:

$$\mu_{NN} = \lambda(\gamma)\ |\gamma|^{(n(\gamma)-1)}$$

The equation is applied for shear rates $\gamma \geq 0.1\ 1/s$ and includes the shear-dependent factors $\lambda(\gamma)$ and $n(\gamma)$:

$$\lambda(\gamma) = \mu_{inf} + \Delta\mu\ \exp(-\left(1+\frac{\gamma}{a}\right)\ \exp(-\frac{b}{\gamma})$$

$$n(\gamma) = n_{inf} + \Delta n\ \exp(-\left(1+\frac{\gamma}{c}\right)\ \exp(-\frac{d}{\gamma})$$

For the approximation of the blood properties, following parameters were used:

μ_{inf}:	3.5 mPa s	n_{inf}:	1
$\Delta\mu$:	25 mPa s	Δn:	0.45
a:	70.71 1/s	c:	70.71 1/s
b:	4.24 1/s	d:	5.66 1/s

S5: Wall shear stress calculation

Wall shear stress (WSS, τ_ω) is calculated by CFD-Post with the following equations:

$$\tau_\omega = \rho\ u^*\ u_\tau$$

In this equation, ρ is the density, u^* an alternative velocity scale in the logarithmic near-wall region and u_τ the friction velocity. The latter two are calculated as follows:

$$u^* = C_\mu^{1/4}\ k^{1/2}$$

with C_μ and k being a k-ε model constant and variable in the SST turbulence, respectively, and

$$u_\tau = \frac{U_t}{\frac{1}{\kappa}\ \ln(y^*) + C}$$

with U_t being the velocity tangent to the wall at the distance Δy from the wall, κ the Karman constant, C a wall-roughness-dependent log-layer constant and y^* the dimensionless u^*-associated wall distance, defined by

$$y^* = \frac{\rho\ u^*\ \Delta \mathrm{y}}{\mu}$$

with μ being the dynamic viscosity.
